# Candidate genes that have facilitated freshwater adaptation by palaemonid prawns in the genus *Macrobrachium*: identification and expression validation in a model species (*M. koombooloomba*)

**DOI:** 10.7717/peerj.2977

**Published:** 2017-02-08

**Authors:** Md Lifat Rahi, Shorash Amin, Peter B. Mather, David A. Hurwood

**Affiliations:** 1 Science and Engineering Faculty, School of Earth Environment and Biological Sciences, Queensland University of Technology (QUT), Brisbane, Queensland, Australia; 2 Science and Engineering Faculty, School of Biomedical Sciences, Queensland University of Technology, Brisbane, Queensland, Australia

**Keywords:** *Macrobrachium koombooloomba*, Freshwater adaptation, ALD, Endemic prawn

## Abstract

**Background:**

The endemic Australian freshwater prawn, *Macrobrachium koombooloomba*, provides a model for exploring genes involved with freshwater adaptation because it is one of the relatively few *Macrobrachium* species that can complete its entire life cycle in freshwater.

**Methods:**

The present study was conducted to identify potential candidate genes that are likely to contribute to effective freshwater adaptation by *M. koombooloomba* using a transcriptomics approach. *De novo* assembly of 75 bp paired end 227,564,643 high quality Illumina raw reads from 6 different cDNA libraries revealed 125,917 contigs of variable lengths (200–18,050 bp) with an N50 value of 1597.

**Results:**

In total, 31,272 (24.83%) of the assembled contigs received significant blast hits, of which 27,686 and 22,560 contigs were mapped and functionally annotated, respectively. CEGMA (Core Eukaryotic Genes Mapping Approach) based transcriptome quality assessment revealed 96.37% completeness. We identified 43 different potential genes that are likely to be involved with freshwater adaptation in *M. koombooloomba*. Identified candidate genes included: 25 genes for osmoregulation, five for cell volume regulation, seven for stress tolerance, three for body fluid (haemolymph) maintenance, eight for epithelial permeability and water channel regulation, nine for egg size control and three for larval development. RSEM (RNA-Seq Expectation Maximization) based abundance estimation revealed that 6,253, 5,753 and 3,795 transcripts were expressed (at TPM value ≥10) in post larvae, juveniles and adults, respectively. Differential gene expression (DGE) analysis showed that 15 genes were expressed differentially in different individuals but these genes apparently were not involved with freshwater adaptation but rather were involved in growth, development and reproductive maturation.

**Discussion:**

The genomic resources developed here will be useful for better understanding the molecular basis of freshwater adaptation in *Macrobrachium* prawns and other crustaceans more broadly.

## Introduction

Understanding evolutionary adaptive processes is important in neoteric biological sciences because it can help to explain the staggering biodiversity currently present on earth. While much progress has been made in this regard, recent development of new genomic techniques have allowed significant advances to occur in this regard. There are numerous species in nature with many interesting traits and effective colonization of freshwater by marine taxa is undoubtedly one of the processes that has contributed significantly to extant levels of biodiversity on earth. Of the approximately 26 multicellular animal phyla that originated in the sea, 15 to date have been able to colonize freshwater later during their evolution (Vogt, 2013). Crustaceans in particular, are well known for their relative success in colonizing freshwater habitats from the marine environment (Vogt, 2013; Moshtaghi et al., 2016). This process is thought to be still ongoing with various taxa having evolved a wide diversity of osmoregulatory strategies to enable this transition (Ordiano, Alvarez & Alcaraz, 2005; Freire, Onken & McNamara, 2008). Important features that have made crustaceans ideal candidates for exploring freshwater adaptation include; huge species diversity (67,000 extant species of which 32% occupy freshwater), relatively large body size, wide habitat diversity, evidence for adaptive physiological traits, a natural monophyletic grouping, diverse osmoregulatory patterns and no taxa have reinvaded marine systems (Vogt, 2013). Moreover, many species still transit regularly between fresh and brackish/marine water which is considered an important step in the evolution of successful obligate freshwater species (Betancur-R et al., 2012).

Interesting traits that have evolved in freshwater crustaceans during the process of freshwater colonization and adaptation include: reduction in relative fecundity with consequent changes to egg size, extension of brood care (prolonged maternal gestation period of larvae), abbreviation of larval developmental stages, and lecithotrophic eggs to increase offspring fitness and survival in new environments (Vogt, 2013). This is because colonization of a new environment is always challenging and offspring are often the most severely impacted by the transition. Thus, crustaceans that inhabit low ionic freshwater environments have evolved a variety of traits that facilitate larvae experiencing increased contact with the surrounding medium prior to hatching. The major obstacle during freshwater adaptation is to deal with osmotic stress and to restrict ion loss from the body in a dilute medium (McNamara et al., 2015). The crustacean gill is well known to play a principal role in osmoregulation and maintenance of ionic balance while the antennal gland (main excretory organ and used to absorb ions) is also known to have important functions under freshwater conditions (Henry et al., 2012). Major challenges crustaceans face to efficiently osmoregulate in freshwater include; absorbing ions from food and the surrounding water, while at the same time minimizing ion loss through semi-permeable membranes (Freire, Onken & McNamara, 2008; Furriel et al., 2010). Temperature shock and change in food habit are additional challenges that organisms may need to deal with during freshwater invasion and colonization (Betancur-R et al., 2012). Freshwater crustaceans thus, need to spend substantially more energy for ionic balance than comparable marine species (Pequeux, 1995; Amado, Freire & Souza, 2006; Moshtaghi et al., 2016). Moreover, regular requirement for moulting to grow potentially exposes extremely soft and vulnerable tissues to the surrounding environment so, maintaining ionic balance is more difficult. These characteristics clearly indicate that freshwater can be considered to be a difficult and challenging environments to colonize.

The physiological and mechanistic aspects of freshwater adaptation involve active ion uptake and absorption, reduction of ion loss and simultaneous regulation or maintenance of cell volume to avoid water gain (McNamara & Faria, 2012). The genetic/genomic basis of this process is mediated by changes in gene expression pattern to cope with the surrounding medium (Wray, 2013). To date, however, we know very little about the molecular basis of this process. Ontogenetic patterns in crustaceans indicate that not all tissues and/or organs develop fully during earlier stages of individual development (Henry et al., 2012). The main osmoregulatory tissue (gill) in crustaceans does not exist/develop completely during earlier life history stages (larvae); branchiostegites located in the branchial chamber provide for osmoregulation and only in later developmental stages the chamber is replaced by the gill (Henry et al., 2012). Thus, we might expect to see changes in gene expression patterns of osmoregulatory genes at different life history stages in crustaceans.

Maintenance of ion balance (osmoregulation) is the most important (and probably the main) mechanism that has allowed freshwater adaptation but some other important and support mechanisms (cell volume regulation, change in body fluid or haemolymph concentration and maintaining cell junctions) may also contribute. So far, osmoregulatory patterns have been widely studied in a diverse array of aquatic species to better understand freshwater adaptation but less attention has been paid to these ancillary processes. Modern genomic technologies can offer important tools for exploring many ecological and evolutionary questions.

Palaemonid prawns are one of the most diverse and widespread decapod crustacean groups that have invaded and colonized freshwater successfully many times from marine ancestors. Extant species show varying degrees of adaptation to low saline environments and possess a wide diversity of osmoregulatory traits. Understanding the molecular basis and ontogeny of this process, therefore, is of great interest to evolutionary biologists. At present, one of the most species rich crustacean groups is the genus *Macrobrachium* that includes 258 described species worldwide and have been broadly categorized into two ecological groups: ALD (abbreviated larval development) and ELD (extended larval development) types, where ELD species require brackish or sea water to complete larval development and this process potentially involves many larval developmental stages (6–14) for up to 2–12 weeks or longer (Jalihal, Sankolli & Shenoy, 1993; Wowor et al., 2009; McNamara et al., 2015). To date, only 25 ALD *Macrobrachium* species have been recognized globally, while all remaining species are recognized as ELD species (Vogt, 2013). Molecular studies support the origin of *Macrobrachium* species through multiple freshwater invasions from marine ancestors (at least nine independent invasions across all continents except Antarctica) (Murphy & Austin, 2005; Liu, Cai & Tzeng, 2007; Wowor et al., 2009; Pileggi & Mantelatto, 2010). *Macrobrachium koombooloomba* in this regard undoubtedly provides an ideal candidate species to investigate successful colonization of freshwater habitat. This is because this species completes its entire life cycle in pure, low ionic freshwater conditions. Knowledge of the molecular basis of successful colonization of freshwater can provide a reference point for examining the evolution of various physiological, behavioral, and ecological traits in other species that have not reached this stage of freshwater adaptation.

*M. koombooloomba* is an endemic Australian freshwater Palaemonid species that has a distinctive and geographically extremely restricted distribution (Short, 2004). This species is confined to a single catchment in the upper Tully River of Northern Queensland (Bernays et al., 2015). Like a limited number of *Macrobrachium* taxa, *M*. *koombooloomba* does not require brackish or sea water to complete their larval development (Short, 2004; Wowor et al., 2009). Unlike most palaemonid prawns, fecundity in this species is extremely low (only 10–70 eggs per brood) but eggs are of relatively very large size and females gestate eggs for a comparatively long period of time (Short, 2004). Thus, specific life history traits of *M*. *koombooloomba* include an ALD or direct larval development, i.e., have a single larval developmental stage and newly hatched offspring are considered to be post larvae, and are immediately well adapted to the surrounding medium (Short, 2004).

Recent molecular studies based on neutral mitochondrial genes (CO1 and 16S) confirm that *M*. *koombooloomba* is most closely related to another endemic Australian freshwater prawn (but one that is widely distributed across the country), *M*. *australiense* (Short, 2004; Murphy & Austin, 2005; Bernays et al., 2015). While *M*. *australiense* is classified as an ALD species, adaptation to obligate freshwater conditions has not developed to the same degree as in *M*. *koombooloomba* because *M. australiense* populations can utilize brackish water across much of its natural distribution. *M*. *koombooloomba* therefore, provides a model crustacean that possesses unique attributes for understanding the molecular basis of freshwater adaptation in this genus. Unfortunately, there is little or no available information on various physiological (including osmoregulation, salinity tolerance etc.) and genetic/genomic aspects of this species. Genomic characterization of genes that uniquely allow *M. koombooloomba* to utilize pure freshwater environments across their entire life cycle can be used as a starting point to help to understand the generalized pattern of the role of different candidate genes and associated molecular processes that allow freshwater adaptation in different crustacean lineages. In particular, a transcriptomics approach can help us to identify all of the genes that are important to a freshwater lifestyle because *M*. *koombooloomba* is well-adapted to a totally freshwater environment, and all of the genes involved with this process should be highly expressed. This analysis can also permit us to examine which genes are important at different life history stages (between larvae, juveniles and adults), and how they produce their functional traits. The principal aim of this study therefore, was to identify the key candidate genes involved with freshwater adaptation in an obligate freshwater prawn species (*Macrobrachium koombooloomba*) using a transcriptomic approach to better understand the molecular genomic processes involved.

## Materials and Methods

### Sample Collection and preservation

A number of individuals (a total of 28 individuals: seven post larvae, nine juveniles and 12 adults) of the target freshwater prawn species (*M. koombooloomba*) were caught using bait traps from Carpenter (17°44′57.9″S 145°37′10.6″E) and Carron (17°45′02.5″S 145°36′21.0″E) Creeks (tributaries of the upper Tully River) in North Queensland, Australia in October 2014. Field permit was issued by the Department of Agriculture, Fisheries and Forestry, Queensland Government (Permit number: 166312). Immediately after collection, individuals were dissected *in situ* to obtain target tissues. Gill, antennal gland, eye stalk and intestine muscle tissues from adult and juvenile prawns, and the whole body of post larvae (PLs) were dissected into small pieces and samples preserved instantly in RNAlater^®^ solution (Applied Biosystems, Warrington, UK). Each PL was preserved in separate tubes with RNAlater. Remaining body parts from juveniles and adults were preserved in absolute ethanol. Water temperature, salinity and conductivity at sampling sites were 19–20 °C, 0‰ and 200–221 µS/m, respectively. Dissected tissues in RNAlater^®^ solution were kept at room temperature for 24 h and then brought to the Molecular Genetics Research Facility (MGRF) at the Queensland University of Technology (QUT) where the samples were preserved at −80 °C temperature prior to RNA extraction.

### RNA extraction, cDNA library preparation and Illumina deep sequencing

Prior to RNA extraction, tissues were removed from RNAlater^®^ solution. Due to the small amount of tissue available per individual, different tissues for each adult and juvenile individuals were pooled together while all dissected tissues of PLs were used for RNA extraction. Tissue materials from different individuals were then crushed in liquid nitrogen to produce a fine powder to maximize RNA yield. Total RNA from all samples were extracted by homogenization and cell lysis using a TRIzol/chloroform extraction method (Chomczynski & Mackey, 1995) and samples then purified using an RNeasy Mini Kit (Cat # 74104, QIAGEN, Germany) according to the manufacturer’s protocol. Total RNA samples were then digested using a TURBO DNA-free™ kit (Ambion, Life Technologies) to obtain DNA-free total RNA. Total RNA quality and yield quantity were checked via 2% agarose gel electrophoresis, Nano Drop 2000 Spectrophotometer (Thermo Scientific) and Bioanalyzer with RNA nano-chip (Agilent 2100, version 6). Purified and DNA digested total RNA samples were then preserved at −80 °C prior to cDNA library preparation.

All of the collected prawns were used for RNA extraction while the higher quality (higher RNA concentration) six samples were used in subsequent steps (cDNA library preparation and sequencing). For mRNA isolation and purification, 4 µg of total RNA was used as the starting material. An Illumina TrueSeqv1 RNA sample preparation kit (Illumina, San Diego, USA) was used for poly-A mRNA capture and purification using oligo (dT) primed magnetic beads. Purified mRNA was then fragmented chemically into smaller fragments and converted to double stranded cDNA using random hexamer primers. Synthesized cDNA fragments were then ligated to Illumina paired end sequencing adapters followed by an end-repair step. Sequencing adapters contain sample specific barcoding sequences that allow sequencing of multiple cDNA libraries on a single lane of an Illumina flow-cell. In order to obtain the final libraries, cDNA fragments were purified, size selected on a gel and amplified via PCR. The cDNA library preparation involved several capture and washing steps using magnetic beads, ethanol and re-suspension buffers. In total, six cDNA libraries were prepared (two for post larvae, two for juveniles and two for adults). The quality and concentrations of each cDNA library was assessed using Bioanalyzer (Agilent 2100, version 6), Qubit^®^2.0 Fluorometer (Invitrogen, Life Technologies) and RT-qPCR (XXPress Thermal Cycler, BJS Biotechnologies, UK). Different dilutions were used for library normalization and then equal quantities of libraries were pooled into a single aliquot for final sequencing (Nakasugi et al., 2013). All cDNA libraries were sequenced on an Illumina NextSeq™ 500 Platform (Illumina, San Diego, USA) at MGRF, QUT and this resulted in more than 227 million (227,564,643) copies of 75 nt paired end high quality reads.

### Quality filtering and *de novo* sequence assembly

Illumina paired end reads were trimmed to remove sequencing adapters using Trimmomatic software (Bolger, Lohse & Usadel, 2014). The quality of Illumina raw reads was checked using FastQC software (Andrews, 2010). Reads were further processed in Trinity assembler supported Trimmomatic software program using a Perl script with default settings that included SLIDINGWINDOW 4:20, MINLEN: 36 (scan the reads with 4 base sliding window and trim when average quality per base falls below 20, avoid reads below 36 bases in length) options (Haas et al., 2013; Bolger, Lohse & Usadel, 2014). High quality raw reads (*Q* ≥ 20 , Phred score above 20) were used for *de novo* assembly using Trinity assembler in order to generate longer contigs. All quality filtered cDNA libraries were pooled together for *de novo* assembly to generate a reference transcriptome.

### Assessment of transcriptome assembly completeness

CEGMA (version 2.5) software package was used to assess the completeness of *de novo* assembly by finding orthologs of core proteins in the sequences (Parra, Bradnam & Korf, 2007). This software package defines sets of conserved protein families and provides a unique mapping procedure to accurately identify exon-intron structures in the sequences.

### Blasting, mapping and functional annotations

The assembled contigs were split into many small files (total 84 smaller files for 125,917 contigs, each file contained approximately 1,500 contigs) that were then blasted in parallel against the NCBI non redundant (nr) database using Basic Local Alignment Search Tool (BLAST+, version 2.2.29) applying a stringency *e* value of ≤1 e^−6^ to find homologous/orthologous genes (Camacho et al., 2009). The resulting blasted sequence contig files were loaded subsequently in to the Blast2GO Pro software suite for mapping and gene ontology (GO) term annotations. GO terms were assigned to contigs that received significant BLAST hits with protein function information and these contigs were then used for InterPro scanning. GO terms and InterPro scan IDs for the assembled contigs were derived following sequence motif searching.

### Protein domain identification and functional enrichment analysis

Assembled contigs generated in Trinity were used to extract protein coding regions using TransDecoder software (Brekhman et al., 2015) that yielded the longest ORF (open reading frame) for each contig. The output files were then used for blasting against the Pfam protein database using HMMER (Finn, Clements & Eddy, 2011; Reumont et al., 2014) searches for protein domain identification. Extracted protein domains were then investigated carefully to find novel transcripts or transcripts of interest. Functional enrichment (gene set enrichment) analysis was performed using the Trinotate software package (Brekhman et al., 2015). For this analysis, assembled contigs were blasted against three different protein databases: swissPort, Uniref90 and Pfam. All GO assignments were then extracted for each gene feature including all parent terms within the GO, using a Perl script in Trinotate software. The final enrichment analysis results (enriched and depleted) were obtained from the software package Bioconductor GOSeq at FDR ≤ 0.001 (Young et al., 2010).

### Identification of candidate genes

Major candidate genes involved with ion regulation and osmoregulation (considered to be the major mechanism for freshwater adaptation) identified in other relevant species were listed following a detailed literature survey (Table S1). Both the transcriptome and protein domain data sets were examined carefully to identify genes (transcripts) involved with the target phenotype (freshwater adaptation) based on BLAST hit matching. Candidate genes were identified based on GO terms related to ion exchange, haemolymph, cellular junction, cell volume, egg size control and larval developmental patterns from the literature survey and BLAST sequence description. Sequences of identified genes were then further blasted against NCBI (National Centre for Biotechnology Information) non redundant (nr) protein database to assess similarity with previously identified and annotated genes in other crustacean species. Following this initial validation step, additional 5′-3′ un-translated regions (UTRs) and open reading frames (ORFs) were detected using the online program ORF Finder. Sequences in the ORF region were then translated into protein to compare both the protein and nucleotide sequences of the candidate genes manually against GenBank data sets.

### Transcript abundance estimation and differential gene expression (DGE) profile

DGE profiles were compared for post larvae (PL), juveniles and adults using Perl scripts in edgeR Bioconductor supported by Trinity (Haas et al., 2013). High quality raw reads from each library were mapped against the assembled reference transcriptome for the DGE analysis. Initially, transcript abundance was estimated using RSEM (RNA-Seq via Expectation-Maximization) that provides expression value matrices (by calculating maximum likelihood abundance estimation at 95% credibility intervals for genes/isoforms) for each library (Nakasugi et al., 2013). The abundance estimation process in RSEM involves 2 steps: estimation of the number of fragments that can be derived from an isoform or gene (also known as expected counts, EC), and the estimated fragment of transcripts within the sample that is represented by the given isoform/gene. EC values were normalized using the Trimmed Mean of M-values (TMM) normalization method in the script of edgeR in the Trinity package (Nakasugi et al., 2013) to adjust for library size and skewed expression of transcripts. For this method, effective library size of each sample was calculated to normalize EC values but FPKM (Fragments Per Kilobase per Million) values were not calculated. In the second step of RSEM, a TPM (transcripts per million) measure was estimated. The EC and TPM values from each library (sample), basic statistical outputs and residuals were plotted using the edgeR program (Trapnell et al., 2010). TPM value is preferred over the FPKM metric as it is independent of mean expressed transcript length and offers better comparisons between samples (Li & Dewey, 2011). Default parameter settings (*p* value cut off for false discovery rate 0.001) in the edgeR Bioconductor software suite were then used for final DGE analysis for generating output in the form of a heatmap.

**Table 1:** Details of primers used in the present study for the validation of expression pattern of selected genes in *Macrobrachium koombooloomba*.

Gene name	Primer type	Sequence	*T*_*m*_ (°C)	Product Size (bp)
18S	Forward	GCGGTAATTCCAGCTCCA	55.00	200
	Reverse	AGCCTGCTTTGAGCACTCTC	57.60	
Na^+^/K^+^ -ATPase (NKA)	Forward	CCACCCAAACAAACTCCAGA	60.90	226
	Reverse	TCGTGAACTCTTGCTTTCTTG	58.30	
NADH Dehydrogenase (NADH-D)	Forward	TGCCCACAAGACTCATGTTT	59.10	190
	Reverse	TTCCTGGTGGTTCTTCAACA	60.40	
Na^+^/H^+^ exchanger (NHE)	Forward	TTCCTTTTGTCGTCGATGCT	60.80	212
	Reverse	TGATGTTTATCATGTGGTTTAGTGG	60.00	
Tyrosine Phosphatase (TP)	Forward	CCTACCCAGCTGGAGACACT	59.30	202
	Reverse	ACAAGCTCTTCCCCCTCTTC	59.80	
V-type (H^+^) ATPase (VTA)	Forward	TTGGTGCAGTTCCGAGACTT	60.80	218
	Reverse	TTCTCTAACTTCTCAAAGGTTGC	57.00	

### RT-qPCR for validation of gene expression pattern

Full length sequences of potential candidate genes (osmoregulatory, haemolymph maintaining, egg size, cell volume controlling, and larval developmental) for other crustacean species were obtained from the *Daphnia* genome (Colbourne et al., 2011) and GenBank databases for alignment with *M*. *koombooloomba* genes for initial sequence validation. Potential candidate genes were then aligned with the sequences of other species to validate the accuracy of *de novo* assembly using the online alignment program MAFFT (Fu et al., 2012). Geneious 8.1.4 version software (Kearse et al., 2012) was used to design primers (Table 1) for five different genes (two differentially expressed genes between different stages and three osmoregulatory genes that were not differentially expressed) in order to validate the differential expression patterns of transcripts using a RT-qPCR approach. We validated the expression pattern of five different genes and we used the 18S gene as a house keeping (as a reference) gene for the RT-qPCR study. Total RNA (1 µg of total RNA was used) was converted to cDNA by using SensiFAST™ cDNA Synthesis Kit (cat # BIO-65054; Bioline) according to the manufacturer’s protocol. We used the same RNA samples from the pooled tissues (including those that were used for Illumina sequencing) to confirm the validity of DGE analysis. We used RNA from seven individuals from each stage for this validation study. In total, 8 µl of cDNA was used for qPCR step with: 1 µl forward primer, 1 µl reverse primer and 10 µl 2x SensiFAST SYBR No-ROX Mix (cat # BIO-98005). The mix was then placed in the thermal cycler R-Corbett (Model: RG-6000, Australia) under the following conditions: 95 °C for 2 min for polymerase activation and 40 cycles (denaturation at 95 °C for 5 s, annealing at 58–65 °C for 20 s and an extension step at 72 °C for 20 s). A standard melt-curve analysis was performed for each reaction to ensure that a single qPCR product was amplified for each gene. Relative gene expression values were then obtained by normalizing expression values of each candidate genes against the 18S gene using delta-delta method following standard protocols developed by Pfaffi (2001). RT-qPCR results for different genes from different life history stages were compared and analyzed using statistical Software SPSS for testing significant differences at the 5% level of significance.

**Table 2:** Assembly, mapping and annotation statistics for *M*. *koombooloomba* transcriptomics.

Features	Results
Total number of Illumina reads	227,564,643
Number of high quality reads (*Q* ≥ 20)	218,532,638
Total number of assembled contigs	125,922
Mean contig length	799 bp
Median contig length	370 bp
Contig range	200–18,050 bp
N50 value	1,597
Number of contigs blasted	31,272 (24.83%)
Number of contigs mapped	27,686 (21.99%)
Number of contigs annotated	22,560 (17.92%)
Number of contigs with InterPro scan ID	19,176 (15.23%)
Total assembled bases	100,599,021
Transcriptome completeness based on CEGs	96.37%
Illumina reads mapped against reference transcriptome for cDNA libraries	80–91%

## Results

### Sequencing, *de novo* assembly, mapping and functional annotation

The Illumina NextSeq 500 platform yielded 227,564,643 high quality 75 bp paired end raw reads. *De novo* assembly of the highest quality (Q ≥ 20) raw reads resulted in 125,917 contigs, of which 31,272 (24.83%) showed significant BLAST hits. Mean contig length, median contig length, longest contig length and N50 value were 799 bp, 370 bp, 18,050 bp and 1,597, respectively (Table 2). Contigs shorter than 700 bp length had very low BLASTx hit success rates. *Daphnia pulex* was the most common top hit species (Fig. S1), a species that is distantly related to *Macrobrachium koombooloomba* but that is the only crustacean (micro-crustacean) species with a complete and well annotated genome sequence available. Functional annotation analysis of the *M*. *koombooloomba* transcriptome yielded 110,286 gene ontology (GO) terms for 31,272 transcripts, of which 18,575 (59.4%), 6,536 (20.9%) and 6,161 (19.7%) were involved in biological processes, cellular components and molecular functions respectively (Fig. S2). We assessed the *M*. *koombooloomba* transcriptome assembly using CEGMA (Core Eukaryotic Gene Mapping Approach) method to address transcriptome quality and completeness. CEGMA revealed 96.37% of the CEGs (Core Eukaryotic Genes) mapped completely and 97.98% CEGs mapped partially a result that indicates a very complete representation of expressed genes in the *M*. *koombooloomba* transcriptome dataset.

**Figure 1:**
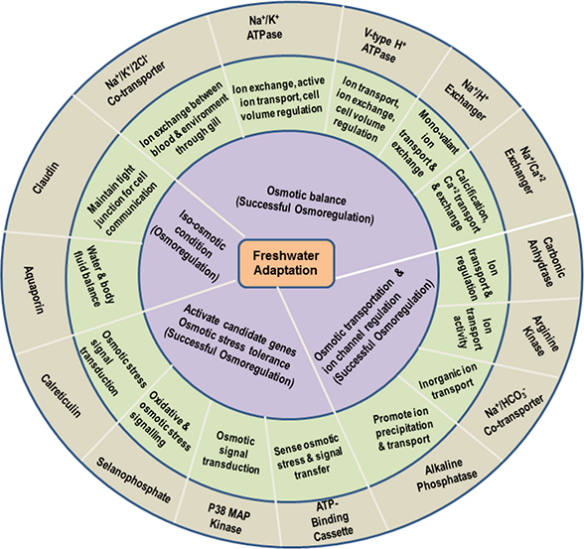
Functional roles of the most important key genes potentially involved in osmoregulation in *M. koombooloomba*. The outermost gray circle represents osmoregulatory genes identified in the present study, the middle green circle identifies the molecular function of candidate genes based on GO terms and the inner blue circle indicates the inferred functional mechanisms that directly lead to freshwater adaptation via maintenance of osmotic balance.

### Identification of candidate genes potentially involved in freshwater adaptation

While the principal mechanism of freshwater adaptation is considered to be maintenance of ionic balance via osmoregulation, other mechanisms that contribute include: cell volume regulation, stress tolerance, water channel regulation, changing and maintaining the body fluid (haemolymph) concentration, changes in egg size and associated changes (abbreviation) in larval development patterns. The *M. koombooloomba* transcriptome yielded 43 different key genes that potentially play a functional role in freshwater adaptation in this species. Figure 1 represents 15 of the most important genes potentially linked to the osmoregulation process in *M*. *koombooloomba* via ion exchange and regulation, signal transfer, molecular transporter and various ion regulatory activities. Genes that are involved with other potential freshwater adaptation mechanisms and genes that play a partial (and/or minor) role in freshwater adaptation in other taxa were also identified in the *M*. *koombooloomba* transcriptome and are listed in Table 3. Table 3 also depicts different isoforms of candidate genes with their detailed properties (contig number, contig name, contig length, ORF length and functions of genes/transcripts).

### Abundance estimation and differential gene expression (DGE) profile

**Figure 2:**
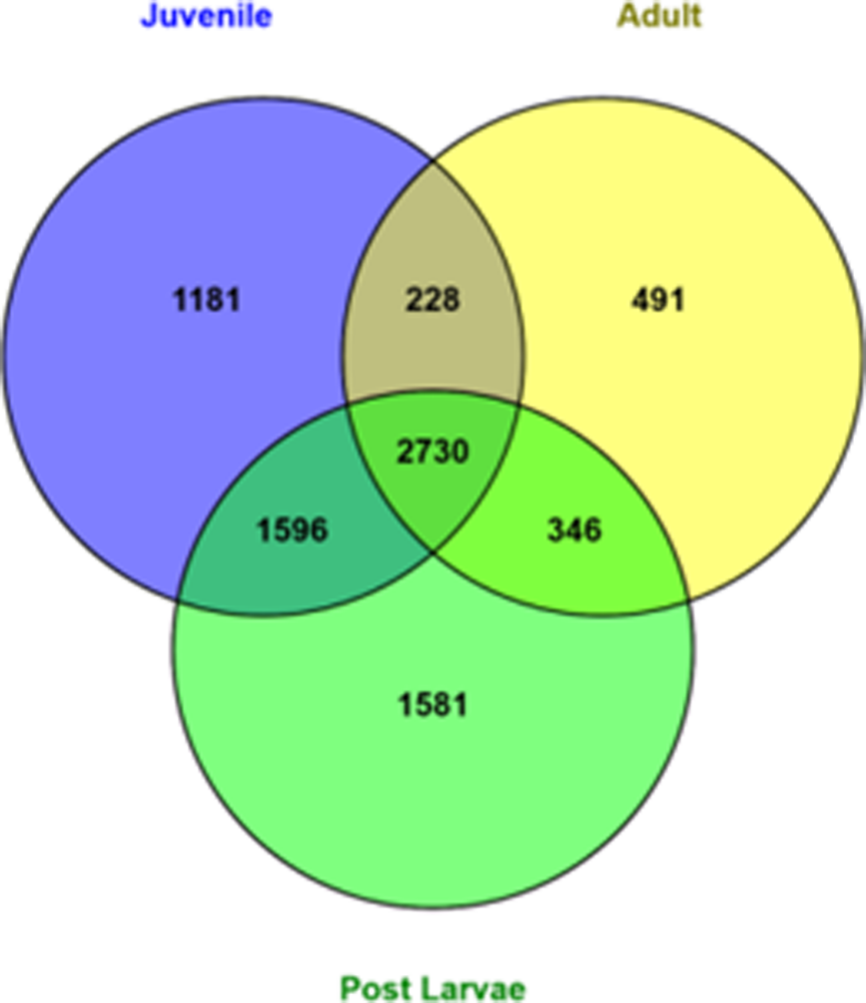
Number of genes expressed at different life history stages in *M. koombooloomba*.

Figure 2 represents the number of genes/transcripts expressed in different individual *M*. *koombooloomba* libraries from post larvae, juveniles and adults based on abundance estimation (TPM values). Table S2 shows the top 20 genes expressed in different individuals. The highest numbers (6,253) of expressed transcripts were found in post larvae and the lowest numbers (3,795) were seen in adults. Juveniles also showed quite high numbers (5,753) of expressed transcripts. In total, 2,730 transcripts were found to be common and expressed in all individuals at TPM value ≥ 10.

**Figure 3:**
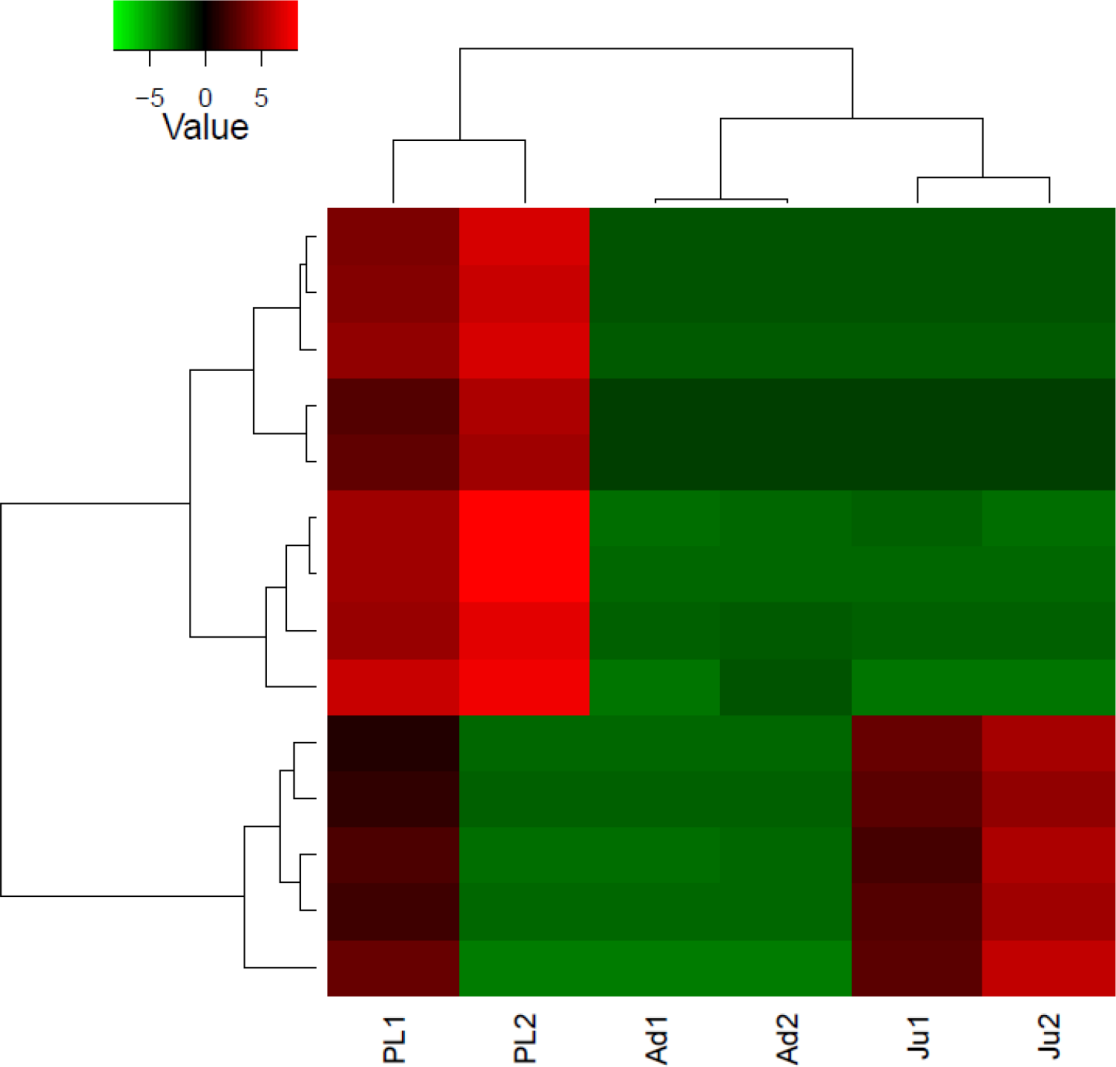
Heatmap showing hierarchical clustering of differentially expressed transcripts (rows) in each sample (column). The red colored transcripts are upregulated while the green colored are the downregulated transcripts. PL1, post larvae 1; PL2, post larvae 2; Ju1, juvenile 1; Ju2, juvenile 2; Ad1, adult 1 and Ad2, adult 2.

Differential gene expression analysis revealed that 15 different transcripts (genes) were expressed differentially in *M. koombooloomba* individuals at different life history stages (Fig. 3). Potential candidate genes that are involved in freshwater adaptation and osmoregulation processes were not however, differentially expressed between the different life history stages. Functional roles of differentially expressed genes were found to include functions in; growth, skeletal and tissue development, and reproductive maturation.

Gene set enrichment analysis revealed no significant differences (at FDR ≤ 0.001) among GO categories for differentially expressed genes; indicating an absence of functionally enriched gene sets (groups of genes that share common biological functions).

**Table 3:** List of identified candidate genes potentially involved in freshwater adaptation in *M. koombooloomba* based on gene ontology (GO) terms from blast hits.

Contig no.	Gene name	Gene function based on GO	Contig length (bp)	ORF (aa)	ORF position
c65624_g1_i1	Alkaline Phosphatase	Phosphatase activity, ion binding and precipitation	2,819	548	383–2,029
c67668_g6_i1	Alkaline Phosphatase 4	Ion binding and de-phosphorylation	2,515	560	755–2,437
c64501_g2_i1	Aquaporin	Ion transport and cell volume regulation	2,467	261	449–1,234
c57042_g1_i1	Aquaporin 10	Integral component of membrane and transporter	5,087	303	3,751–4,662
c66816_g1_i2	Aquaporin 9 isoform 1	Transmembrane and organic substance transport	2,298	318	741–1,697
c66816_g1_i1	Aquaporin 9 isoform 2	Substrate specific membrane transport	1,926	296	741–1,631
c58558_g1_i2	Arginine Kinase	Phosphorylation, ATP binding, salinity regulation	2,880	630	153–2,045
c54350_g1_i1	Arginine Kinase 1	Kinase activity and partial salinity regulation	950	147	334–777
c67563_g1_i1	ABC sub-family a	ATP binding, ATPase activity and ion transport	7,006	2,010	222–6,254
c67774_g1_i2	ABC sub-family b	Transmembrane, heme and some ion transport	7,504	701	50–2,155
c76795_g1_i1	ABC sub-family c	Transferase activity and phosphorus transport	696	201	92–695
c65549_g2_i3	ABC sub-family d	ATP catabolic process, transmembrane transport	8,304	661	6,229–8,214
c8377_g1_i1	Ca^+2^ activated Cl^-^ channel regulator	Protein binding, ion channel activity	808	244	1–734
c56438_g1_i1	Ca^+2^ activated Cl^-^ channel regulator 4	Ca^+2^ and Cl^-^ ion transport, cellular response to hypoxia	3,891	625	1,179–3,056
c68090_g1_i2	Ca^+2^ activated Cl^-^ channel precursor	Ion transport and protein binding	5,339	959	140–3,019
c56147_g1_i1	Ca^+2^-ATPase	Ca^+2^ ion regulation, calcification	3,946	1,020	449–3,511
c50128_g1_i1	Ca^+2^-ATPase serco/endo- plasmic reticulum	Ca^+2^ binding and transport, membrane component, metabolic process, ATP & metal ion binding	4,312	999	229–3,228
c111864_g1_i1	Cbl	Cell signaling, protein ubiquitination, oogenesis	597	185	41–596
c50619_g1_i1	Calreticulin	Signal transduction, ion binding & protein folding	2,067	405	732–1,949
c28633_g1_i1	Calreticulin Precursor	Ca^+2^ homeostasis, salinity regulation under stress	682	222	15–681
c67767_g2_i1	Carbonic Anhydrase	Response to salt stress, ion and protein binding	643	193	62–642
c59766_g1_i2	Carbonic Anhydrase 10	Transferase activity, identical protein binding	2,761	330	1,566–2,558
c50549_g1_i1	*α* -Carbonic Anhydrase	Ion binding and exchange, pH balance	1205	309	177–1,106
c27677_g1_i2	*β* -Carbonic Anhydrase	Ion binding, pH balance & metabolic process	442	62	119–307
c51639_g3_i1	Claudin 2	Bind transmembrane protein, epithelial permeability	2,538	582	668–2,416
c63565_g1_i1	Claudin 3	Establish paracellular barrier, epithelial permeability	4,551	783	25–2,376
c28732_g1_i1	Crustacean cardiovascular peptide	Stress tolerance, signaling and body fluid maintenance	1,247	142	734–1,162
c45921_g1_i1	Crustacean hyperglycemic hormone	Stress signaling pathway, haemolymph production	1,953	135	193–600
c63950_g1_i1	Cullin	Cellular processes, developmental roles	5,010	829	303–2,792
c56596_g1_i1	Diuretic Hormone	Water balance, haemolymph balance	2,552	141	222–647
c42965_g1_i1	Heat shock protein	Response to various stress, chaperone function	2,284	685	158–2,258
c76123_g1_i1	Heat shock protein 70	Stress response, ATP binding, protein folding	2,590	649	152–2,101
c111190_g1_i1	H^+^/Cl^-^ exchanger	Regulate Cl^-^ channel & cell volume, signal transfer	386	91	103–378
c35962_g1_i1	H^+^/Cl^-^ exchanger 7	Ion transmembrane transport & anion regulation	241	70	2–214
c64484_g1_i1	*α* -Integrin	Regulate cell volume & junction in osmotic stress	7,288	1,743	2,023–7,254
c60452_g1_i1	*β* -Integrin	Mediate signal transduction pathway	5,024	823	271–2,742
c60620_g1_i1	ILF2	ATP binding, positive transcriptional regulation	2,255	400	94–1,296
c55508_g2_i1	K^+^Cl^-^ symporter	Integral membrane component, KCl symporter	243	74	21–242
c4158_g1_i1	Leukocyte ARL	Oogenesis, cellular morphogenesis	415	121	49–414
c40058_g1_i1	MAP Kinase	Phosphorylation, osmotic signal transfer, bind ATP	406	99	32–331
c100415_g1_i1	Mastermind	Embryogenesis, development, DNA binding	1,637	375	353–1,480
c55954_g1_i1	Merlin	Developmental role	1,618	176	639–1,169
c67482_g1_i1	Midline	Multiple developmental roles, embryogenesis	2,777	900	30–2,732
c87009_g1_i1	Mitochondrial carrier protein	Anion transport, osmotic signal transduction, transmembrane transport, membrane component	962	57	422–595
c51143_g2_i1	Mothers against DPP 3	Growth transformation signal, metal ion binding	5,766	438	267–1,583
c7394_g1_i1	Mothers against DPP 4	Growth transformation, morphogenesis, gastrulation	241	73	1–222
c64902_g2_i3	Mothers against DPP 6	Organ development,, growth transformation	2,279	148	886–1,332
c52625_g1_i1	Mg^+2^ Transporter	Ma^+2^ transportation, integral membrane component	3,106	326	2,094–3,076
c67333_g3_i3	*α* -Na^+^/K^+^ ATPase	Binds ATP, Ion binding, transport and exchange for osmoregulation, integral membrane component	3,880	1,036	184–3,294
c67333_g3_i2	Na^+^/K^+^ ATPase	Ion exchange & binding, transmembrane transport	3,799	1,009	184–3,213
c49221_g1_i1	*β* -Na^+^/K^+^ ATPase	Ion transport, response stimulus, protein binding	1,452	308	141–1,067
c65989_g1_i2	*β* -Na^+^/K^+^ ATPase 2	Biosynthetic process, ATP binding	2,449	325	1,406–2,383
c45291_g1_i1	Na^+^/HCO _3_ transporter	ATP binding, transport and exchange of anions	1,222	100	794–1,096
c62041_g1_i1	Na^+^/Ca^+2^ exchanger 1	Ca^+2^ transport, Na^+^/Ca^+2^ antiporter activity	4,276	854	1,508–4,072
c63621_g4_i1	Na^+^/Ca^+2^ exchanger 2	Regulate ion transport, Na^+^ import and Ca^+2^ export	2,210	70	839–1,051
c63621_g3_i1	Na^+^/Ca^+2^ exchanger 3	Cell communication, transmembrane transport	4,713	911	259–2,994
c60792_g1_i1	Na^+^/K^+^/2Clcotransporter	Na^+^:K^+^:2Cl^-^ symporter activity, ion transport and exchange, integral membrane component	4,918	1,066	1,716–4,916
c58971_g1_i1	Na^+^/H^+^ exchanger	Na^+^& H^+^ transport, Na^+^:H^+^ antiporter activity	2,687	679	511–2,550
c68501_g1_i1	Na^+^/H^+^ exchanger 2	Cation transmembrane transport, pH regulation	1,809	578	75–1,808
c57971_g1_i3	Na^+^/H^+^ exchanger 3	Integral membrane component, pH regulation	1,201	265	227–1,024
c65672_g1_i1	Na^+^/H^+^ exchanger 7	Na^+^/H^+^ transmembrane transport, Na^+^:H^+^ antiporter	929	98	136–432
c65672_g3_i1	Na^+^/H^+^ exchanger 8	Na^+^/H^+^ transport, pH regulation, antiporter activity	1,417	309	376–1,305
c100508_g1_i1	Na^+^/K^+^/Ca^+2^ exchanger	Ca^+2^/Na^+^:K^+^ antiporter activity, ion transport	238	67	1–204
c66143_g9_i1	Na^+^ transporter	Transmembrane transporter, transporter activity	2,505	552	320–1,978
c61274_g1_i1	Potassium Channel	K^+^ transmembrane transport, K^+^ channel activity	2,246	617	393–2,245
c28820_g1_i1	Selenophosphate	ATP binding, oxidative and salinity stress response	2,186	326	401–1,381
c66394_g3_i1	Serpin	Larval development, chaperone & storage functions	2,391	413	992–2,233
c54043_g1_i1	V (H^+^) ATPase	Pumps H^+^ in dilute medium for ionic balance	3,807	836	262–2,772
c67321_g1_i1	V (H^+^) ATPase 116 kda subunit a	ATP hydrolysis coupled H^+^ transport, integral membrane component, drives osmoregulation	5,208	833	162–2,663
c54413_g1_i1	V (H^+^) ATPase 21 kda subunit c	Producing plasma membrane H^+^ transporting V-ATPase complex, ion exchange, pH balance	1,232	208	121–747
c18864_g1_i1	V(H^+^) ATPase subunit a	Monovalent ion exchange for osmotic balance	2,560	622	593–2,461
c76796_g1_i1	V(H^+^) ATPase subunit b	H^+^ transport and cellular homeostasis	644	103	184–495
c50865_g1_i1	V(H^+^) ATPase subunit d	Biosynthetic process, pH balance, ion balance	1,489	249	590–1,339
c37259_g2_i1	V(H^+^) ATPase subunit e	Cell volume regulation, H^+^ transport	218	61	3–188
c62564_g1_i1	Plekstrin homology domain protein	Intracellular signaling, membrane protein and cellular component	805	150	127–579
c61568_g1_i1	Vitelline membrane outer layer protein	Integral membrane protein	1,297	279	379–1,218
c102973_g1_i1	Vitellogenin	Oogenesis, nutrient reservation and lipid transport	692	198	88–684

### RT-qPCR for the validation of differential gene expression (DGE) pattern

**Figure 4:**
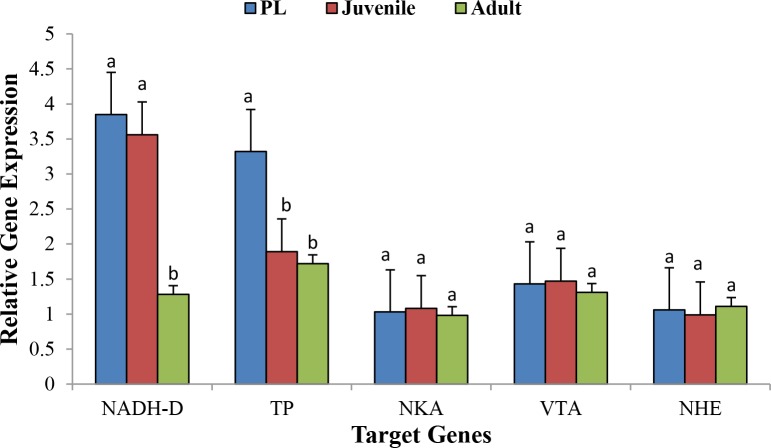
Relative gene expression of five target genes at 3 different life stages of *M. koombooloomba* (including seven biological replicates for each stage). Whole body of PL was used while pooled tissues were used for juvenile and adults. Relative gene expression values of candidate genes were normalized using 18S as a reference gene. Error bars represent ±1 SE.

Figure 4 shows the relative expression pattern of five different genes in different life history stages in *M. koombooloomba*. A significant difference (*p* < 0.05) in expression pattern of the NADH Dehydrogenase gene was observed when PLs and juveniles were compared with adult individuals, but no significant difference was observed between PLs and juveniles. PLs showed a significantly higher expression level over juveniles and adults for the Tyrosine Phosphatase gene. The three osmoregulatory genes (Na^+^∕K^+^-ATPase, V-type H ^+^-ATPase and Na^+^∕H^+^ exchanger) did not show any significant differences in expression patterns among different life history stages. Results confirm the validity of our differential gene expression study.

## Discussion

Transcriptomic data generated in the current study provides an important genomic resource for understanding the functional roles of major candidate genes that contributed to freshwater adaptation in the endemic Australian freshwater prawn species, *M. koombooloomba*. Results also provide a significant genomic resource for a non-model crustacean species for which currently, no transcriptome data are available. Complete freshwater adaptation in crustaceans likely involves genic interactions between seven different major physiological processes including: osmoregulation, cell volume regulation, water channel regulation, stress tolerance, body fluid maintenance, control of egg size and the number of larval developmental stages. In total, 43 candidate genes consisting of a number of isoforms and subunits (Table 3) were identified that potentially play important roles in freshwater adaptation. GO term categories in Table 3 reveal that 25 different gene families were involved directly or indirectly in osmoregulation (including ion exchange and maintenance of ionic balance, ion transport, control of epithelial permeability), five genes were involved with cell volume regulation (regulatory cell volume increase or decrease based on surrounding environment), seven genes were involved in stress tolerance (sensing and signal transduction), eight genes in water channel regulation and cellular epithelial permiability, three genes were involved in maintaining body fluid (haemolymph), nine genes were involved with controlling egg size and number, and three genes in abbreviating larval developmental stages. Furthermore, some of these genes potentially have multiple functions (play roles in different functions/phenotypes simultaneously).

Na^+^∕K^+^-ATPase (NKA) and V-type (H^+^)-ATPase (VTA) are widely considered to be the master genes that control osmoregulation (and/or ion exchange) in aquatic crustaceans because they are directly involved with ion exchange for maintenance of osmotic balance (Genovese et al., 2005; Freire, Onken & McNamara, 2008; Barman et al., 2012; Ali et al., 2015). NKA and VTA are also the most well studied osmoregulatory genes in crustaceans. NKA is considered to be the main gene engaged in maintaining ionic balance in all water types (salinity conditions) but is more active than VTA in osmotic and ionic balance in brackish to saline waters. In low ionic freshwater conditions, VTA has an equally important or more important role compared with NKA (Stillman et al., 2008) but VTA has been less well studied than NKA. Apart from its role in ion exchange and ion balance, VTA also influences cell volume regulation (Barman et al., 2012). Other important genes that influence freshwater adaptation via osmoregulatory process include: carbonic anhydrase (CA), Na^+^∕H^+^ exchanger, Na ^+^/HCO ${}_{3}^{-}$ exchanger, Na ^+^/Ca^+2^ exchanger, Ca^+2^ ATPase, aquaporin, mitogen activated protein (MAP) kinase and alkaline phosphatase (Gao & Wheatly, 2004; Genovese et al., 2005; Berkefeld, Fakler & Schulte, 2010; Nadal, Ammerer & Posas, 2011). MAP kinase is known to be the main gene controlling signal transduction under osmotic, thermal and some other stresses in a wide range of animal species (Nadal, Ammerer & Posas, 2011). A substantial number of studies have been conducted to identify, characterize and to examine expression profiles of NKA, VTA and CA genes in a variety of crustacean taxa (Barman et al., 2012; Pongsomboon et al., 2009; Faleiros et al., 2010; Havird, Henry & Wilson, 2013; Tongsaikling, Salaenoi & Mingmuang, 2013; Ali et al., 2015). There are also other genes that contribute to the osmoregulation process but they play only a minor role. These genes are involved with sensing, signal transduction and ion transport activities (Nadal, Ammerer & Posas, 2011).

It is quite apparent however, that a single gene cannot, in isolation, control a specific phenotype, but rather multiple genes contribute to the trait and/or single genes can have multiple roles in determining several different traits simultaneously (Wray, 2013). While we believe that we have identified virtually all of the main osmoregulatory genes (as listed in Table S1) in the *M*. *koombooloomba* transcriptome dataset, we did not find two other previously identified genes that play a partial (or very minor) role namely: osmotic stress transcriptional genes and cystic fibrosis transmembrane regulator (CFTR). These two genes are known to be involved with tonicity responsive ion channel regulation under hyper-osmotic conditions (Jeoong et al., 2014). As *M*. *koombooloomba* is an obligate freshwater species and does not encounter salt water stress across its life cycle, it is very likely that these genes express at only a very low level in the target species or may have become pseudogenes; and so were not detected in our dataset.

Maintaining body fluid concentration (haemolymph in crustaceans) is crucial for individual survival under various environmental conditions to maintain ionic balance between the intra- and extra-cellular fluids (Sang & Fotedar, 2004; Chen et al., 2016). Genes that are involved with haemolymph production and regulation in crustaceans undoubtedly play a vital role in freshwater adaptation. Here, we identified three different potential candidate genes in the *M*. *koombooloomba* transcriptome data set that are involved with haemolymph production and regulation specifically: crustacean hyperglycemic hormone (CHH), diuretic hormone (DH) and crustacean cardiovascular peptide (CCP) (Chen et al., 2016). In a dilute medium (freshwater), body fluid concentration is higher than the surrounding environment and freshwater inhabitants need to address this problem since they tend to gain water and/or lose ions from their tissues. The three gene families identified above are likely to play key roles in meeting this challenge by regulation haemolymph balance.

Ten genes in *M*. *koombooloomba* were identified that can control egg size and larval development patterns, including: midline, mothers against decapentaplegic (Dpp.), plekstrin, vitelline membrane outer layer protein, vitellogenin, leukocyte-antigen related like, cullin, Cbl, mastermind, merlin and serpin (Table 3). These genes are also likely to be very important genes in the freshwater adaptation process not only in *M. koombooloomba* but also in other crustaceans that are well adapted to freshwater environments. Merlin, Mastermind, Karl and Serpin are the well-known candidate genes that contribute to changes in larval developmental duration in *Drosophila melanogaster* and *Daphnia pulex* (Mensch et al., 2008; Harney, Plaistow & Paterson, 2015). We identified the Merlin, Mastermind and Serpin genes in the *M. koombooloomba* transcriptome but did not find the Karl gene; potentially the Merlin, Mastermind and Serpin genes in *M*. *koombooloomba* could influence larval developmental stages and duration in this species, and thereby, contribute to successful freshwater adaptation. While we did identify mastermind gene in *M*. *koombooloomba*, this gene has not been found in *Daphnia*; potentially indicating that *Daphnia* may have lost this gene during the evolutionary process or via lineage splitting (as gene loss or gain is a common evolutionary process in many taxa) (Colbourne et al., 2011). The karl gene is also absent in *Daphnia* which indicates that the crustacean lineage may have lost this gene during their evolution.

To date, very little information is available about these genes and that which is available comes primarily from some model species. The functional roles of these candidate genes are obviously important in freshwater adaptation because egg size, egg number and number of larval developmental stages are affected by trade-offs via natural selection in freshwater environments (Hancock, Hughes & Bunn, 1998). Unlike many *Macrobrachium* species, *M. koombooloomba* completes its entire life cycle in pure freshwater and the larvae naturally periodically experience fast water flows in small rainforest streams. Larvae are therefore prone to being swept downstream with increasing salinity gradients. To counter this environmental pressure, large egg size and a long gestation period have evolved, such that newly hatched offspring can be considered more post larvae-like, and can swim actively against the current to avoid the problem of downstream displacement. The evolutionary trade-off for producing large sized eggs over a long gestation period to allow larvae to be released is therefore presence of smaller numbers of yolky eggs (Hancock, Hughes & Bunn, 1998).

Invading and colonizing freshwater from a marine ancestral state will likely involve evolution of a significant stress tolerance capacity. Different candidate genes that respond immediately to provide support for stress mitigation under changed osmotic conditions to date include: selanophosphate, heat shock proteins (HSP), calreticulin, MAP kinase, ABC proteins and various molecular transporters (Genovese et al., 2005; Nadal, Ammerer & Posas, 2011; Barman et al., 2012). These genes can assist with detection of stress and transfer signals to trigger major changes in the gene expression patterns of candidate genes. Changes only in expression pattern of specific candidate genes however, is probably not sufficient to support colonization of novel osmotic environments under continuous stress. Changes in physiological and phenotypic traits will also be important, and this sometimes includes novel mutations that allow long term adaptation or complete adaptation (Betancur-R et al., 2012; Leite & Zanotto, 2013; Wray, 2013). Changes in genomic architecture (mutation/s in candidate genes and other mechanisms) that allow effective adaptation can take many generations to evolve but once this occurs, they can facilitate successful colonization to the new environment and at the same time, can bring minor to significant changes in some important phenotypic traits (Wray, 2013).

The DGE patterns at different life history stages (post larvae, juvenile and adult) in *M*. *koombooloomba* (Fig. 3) show that key candidate genes influencing osmoregulation, haemolymph production, cell volume regulation, water channel regulation, egg size control and larval development were not expressed differentially (showed similar DGE patterns) in different life history stages. The genes that were expressed differentially in *M*. *koombooloomba* were involved with growth, tissue and skeletal development, and reproductive maturation. RT-qPCR based gene expression study also revealed significant differences for NADH-D (involved with growth and morphogenesis) and TP (involved with skeletal and neural development) genes, but no significant difference (*p* > 0.05) in expression pattern of the three major osmoregulatory genes (NKA, VTA and NHE) in three different life history stages in *M*. *koombooloomba* (Fig. 4). The relative expression levels of these three genes however were quite high (>1 compared to a housekeeping 18S gene), indicating very important functional roles of these genes under freshwater conditions. While all of osmoregulatory and ion balance tissues are not fully developed at earlier life history stages, no significant changes in differential expression pattern of the candidate genes were evident among these stages in *M*. *koombooloomba*. A prolonged gestation period with the mother, apparently must contribute to successful osmoregulation and ion balance control at both the egg and larval stages. Absence of DGE pattern for osmoregulatory genes between three different life stages is highly likely due to the fact that they all inhabit the same natural habitat and are well adapted to this condition. Comparative analysis of the same genes in marine and brackish water *Macrobrachium* taxa would be informative to see if similar patterns were evident or not.

It is quite apparent that freshwater adaptation in crustaceans is a complex process. It is highly likely that this process has involved many genes contributing small effects to the phenotype that in combination can produce phenotypes well adapted to low ionic physical environments. Now that we have potential functional targets to examine in more detail, there is the opportunity to fully understand the genomic basis and processes that have allowed aquatic invertebrates to adapt from marine to freshwater environments.

## Conclusions

Here, we present the first full transcriptome profile for an endemic obligate freshwater-adapted Australian Palaemonid prawn, *Macrobrachium koombooloomba* assembled from Illumina deep sequencing. We identified 43 different key genes families in seven categories that are involved with freshwater adaptation. The most important genes involved in this process include: NKA, VTA, Na^+^∕H^+^-exchanger, MAP kinase, Merlin, Selanophosphate, Calreticulin, Vitellogenin, Cullin, Cbl, Serpin, DH, CCP and CHH. Results indicate that control of osmoregulatory capacity is not the sole mechanism that contributes to freshwater adaptation in crustacean taxa. Complete adaptation to freshwater environments likely involves a combination of various complex arrays including: maintenance of ionic balance via osmoregulation, cell volume regulation/control, water channel regulation, stress tolerance ability, production and maintaining body fluid for water balance, changes to egg size and number and changes to larval developmental pattern. Currently, little is known about the genes that control the freshwater adaptation process in other crustacean lineages. Our data can provide an important foundation for developing more specific transcriptome to phenome comparative studies of Palaemonid taxa that have reached various stages in the transition from marine to freshwater environments (genomic basis of single and multiple traits evolution and/or diversity in closely related species or distant species with shared biological traits).

##  Supplemental Information

10.7717/peerj.2977/supp-1 Table S1 Key candidate genes involved with osmoregulation in prawns and other aquatic crustacean species

10.7717/peerj.2977/supp-2 Table S2 Top 20 transcripts expressed at different stages in *M. koombooloomba*

10.7717/peerj.2977/supp-3 Figure S1 Top hit species distribution chart X-axis represents the number of blast matches while Y-axis shows the name of different species.

10.7717/peerj.2977/supp-4 Figure S2 WEGO plot showing number and percentage of genes involved with different functions

## Additional Information and Declarations

### Field Study Permissions

The following information was supplied relating to field study approvals (i.e., approving body and any reference numbers): Field permit was issued by the Department of Agriculture, Fisheries and Forestry under Queensland Government Permit Number: 166312.

### Data Availability

The following information was supplied regarding data availability: Rahi, Lifat (2016): Illumina Forward sequences for Adult and Juvenile Individuals.
https://dx.doi.org/10.6084/m9.figshare.3753402.v1. Rahi, Lifat (2016): Reverse Sequences for Adult and Juvenile Individuals. figshare.
https://dx.doi.org/10.6084/m9.figshare.3753531.v1. Rahi, Lifat (2016): Forward Illumina Sequences or Post Larvae. figshare.
https://dx.doi.org/10.6084/m9.figshare.3753528.v1. Rahi, Lifat (2016): Reverse Illumina Sequences of Post Larvae. figshare.
https://dx.doi.org/10.6084/m9.figshare.3756438.v1.
