# Supplementary material for: Candidate genes that have facilitated freshwater adaptation by palaemonid prawns in the genus Macrobrachium: identification and expression validation in a model species (M. koombooloomba)
Source: PeerJ. 2017 Feb 8;5:e2977. doi: 10.7717/peerj.2977 (PMC5301973; doi:10.7717/peerj.2977)
Supplement: Table S1 [file peerj-05-2977-s001.pdf]

Table S1: Key candidate genes involved with osmoregulation in prawns and other aquatic crustacean species.

Gene Name	Functions and Mechanisms	Species Studied
Na^+/K^+- ATPase (NKA)	The most important (master) gene for osmoregulation, exchange ions depending on external medium, establish electromechanical gradients across gill membranes	Crayfish (Ali et al., 2015), *M. amazonicum* (Faleiros et al., 2010), *M. australiense* (Moshtaghi et al., 2016)
V type (H^+) ATPase (VAT)	Drives osmoregulation in dilute/extreme freshwater medium, pump protons for overall ion exchange	*M. amazonicum* (Faleiros et al., 2010), *M. australiense* (Moshtaghi et al. 2016)
$Na^+/K^+/2Cl^-$cotransporter (NKCC)	Ion transportation into gill cells either from the blood or environment depending on salinity of external medium	*M. australiense* (Moshtaghi et al., 2016), Crayfish (Ali et al., 2015)
Carbonic Anhydrase (CA)	Produce H^+& HCO_3^- to drive Na^+& Cl^- exchange	*M. australiense* (Moshtaghi et al. 2016)
Alkaline Phosphatase	Ion precipitation, support Ca^{+2}- ATPase for calcification	Mud crab (Tongsaikling et al., 2013)
Arginine Kinase	Support ion transport and partial role in salinity regulation	*M. australiense* (Moshtaghi et al. 2016)
Calreticulin	Signal transduction, Ca^{+2} homeostasis, salinity stress biomarker, oxidative stress response and molecular chaperon	*M. rosenbergii* (Barman et al., 2012), crayfish (Ali et al., 2015)
CFT Regulator	Cl^- channel regulator for euryhaline animals	Crayfish (Havird et al., 2013)
ABC (ATP-binding cassette) C12 protein	Participate in ion channel regulation and osmoregulatory pathway, significant role in salinity adaptation in gill	*M. rosenbergii* (Barman et al., 2012)
Ca^{+2}- ATPase	Ca^{+2} transport & exchange, calcification, Ca^{+2}homeostasis	Crayfish (Gao and Wheatley, 2004)
Na^+/H^+ exchanger	Exchange of Na^+ for H^+ or vice versa, cell volume regulation	*M. amazonicum* (Falaeiros et al., 2010)
Na^+/HCO_3^-cotransporter	Transport HCO_3^- and exchange $HCO_3^-$for Na^+	Green crab (Havird et al., 2013)
H^+/Cl^- exchanger	Exchange of H^+ for Cl^- or vice versa depending on salinity	Crab (Genovese et al., 2013)
Na^+/Ca^{+2} exchanger	Remove Ca^{+2} from cell and uptake Na^+ from environment	Crayfish (Gao and Wheatley, 2004)
Ca^{+2} activated K^+ channel	Signal transduction for Ca^{+2} transport and other cations	Lobster (Berkefeld et al., 2010)
$Ca^{+2}Cl^-$ channel regulator	Exchange of 1 Ca^{+2} for 2 Cl^- for ion regulation	Crayfish (Havird et al., 2013)
Cl^- transporter	Transportation of Cl^-	Crayfish (Havird et al., 2013)
Cl^-/HCO_3^- exchanger	Catalyze HCO_3^- from inside the cell for Cl^- outside the cell	*M. australiense* (Moshtaghi et al. 2016)
Mg^{+2} transporter	Mg^{+2} transport across cell membrane & signal transduction	Crab (Leite and Zanotto, 2013)
K^+Cl^- symporter	Transport K^+& Cl^- ions, maintain electrochemical balance	*P. monodon* (Pongsomboon et al., 2009)
Integrin	Supporting role to control homeostasis for salinity stress	*M. rosenbergii* (Barman et al., 2012)
Claudin 3	Maintain epithelial permeability, sensing osmotic stress	Freshwater crab (Furriel et al., 2010)
Aquaporin 3	Regulate cell volume caused by osmotic stress	*M. australiense* (Moshtaghi et al. 2016)
P38 MAP kinase	Signal transduction for stress response to osmotic balance	*M. rosenbergii* (Barman et al., 2012)
Osmotic Stress transcription 1	Signal transduction during osmotic stress	*M. rosenbergii* (Barman et al., 2012)
Mitochondrial carrier protein	Osmoregulatory signal transduction	Crab (Genovese et al., 2005)
Selenophosphate 1 (SPS1)	Tolerance of oxidative and salinity fluctuation stresses	*M. rosenbergii* (Barman et al., 2012)
ILF2 (interleukin enhancer binding factor 2)	A transcriptional regulator involved in the physiological process of euryhalinity	*M. australiense* (Moshtaghi et al. 2016) *M. rosenbergii* (Barman et al., 2012)
