# Supplementary material for: Candidate genes that have facilitated freshwater adaptation by palaemonid prawns in the genus Macrobrachium: identification and expression validation in a model species (M. koombooloomba)
Source: PeerJ. 2017 Feb 8;5:e2977. doi: 10.7717/peerj.2977 (PMC5301973; doi:10.7717/peerj.2977)
Supplement: Table S2 [file peerj-05-2977-s002.pdf]

Table S2: Top 20 transcripts expressed at different stages in *M. koombooloomba*.

Post Larvae			Juvenile			Adult		
**Transcript ID**	**Description**	**TPM**	**Transcript ID**	**Description**	**TPM**	**Transcript ID**	**Description**	**TPM**
c898_g2_i1	Ribosomal gene	156,729	c898_g2_i1	Ribosomal gene	198,613	c898_g2_i1	Ribosomal gene	147,463
c67584_g2_i1	α-Hemocyanin	15,104	c56075_g1_i1	Cysteine	15,968	c55870_g1_i1	Claw keratin protein	21,368
c32579_g1_i1	Male reproductive	11,658	c55019_g2_i1	Cystinosin homolog	13,714	c62394_g3_i1	Cuticle proprotein	14,932
c67933_g2_i1	Actin	10,956	c67811_g1_i1	Opsin	13,210	c56075_g1_i1	Cysteine	12,886
c56365_g1_i1	Myosin light chain	10,300	c898_g1_i1	NADH 1	11,101	c61841_g2_i1	Calcification peptide	12,107
c62482_g3_i1	Cathepsin I	9,860	c54442_g1_i1	Hypothetical protein	10,990	c57061_g1_i1	Unidentified protein	10,334
c61841_g2_i1	Calcification peptide	9,418	c60131_g1_i1	Mitochondri al carrier	10,575	c55019_g2_i1	Cystinosin homolog	10,038
c56075_g1_i1	Cysteine	9,358	c50494_g1_i1	Neuron protein	10,433	c54442_g1_i1	Hypothetical protein	9,711
c84130_g1_i1	Cuticle protein	9,222	c55019_g1_i1	α-ATP synthase	9,115	c54430_g2_i1	Antisense protein	9,509
c55019_g2_i1	Cystinosin homolog	8,333	c57643_g1_i1	Antisense protein	8,372	c60131_g1_i1	Mitochondri al carrier	8,643
c55034_g2_i1	Growth protein	8,130	c92391_g1_i1	Cystisolic protein	7,833	c47187_g1_i1	Ca^{+2} binding protein	8,103
c47187_g1_i1	Ca^{+2} binding protein	7,979	c62348_g3_i1	α-Elongation factor	7,594	c55019_g1_i1	α-ATP synthase	8066
c54442_g1_i1	Hypothetical protein	7,777	c108471_g1_i1	NADH dehydrogena se	7,084	c67811_g1_i1	Opsin	7428
c60131_g1_i1	Mitochondrial carrier	7,536	c47187_g1_i1	Ca^{+2} binding protein	6,535	c67933_g2_i1	Actin	7266
c61308_g2_i1	Growth arrest protein	7,367	c67933_g2_i1	Actin	6,504	c50361_g2_i1	Unidentified protein	7,262
c55870_g1_i1	Claw keratin protein	7,281	c62464_g1_i1	β-ATP synthase	5,356	c50494_g1_i1	Neuron protein	7,254
c55019_g1_i1	α-ATP synthase	6,576	c61841_g2_i1	Calcification peptide	5,337	c84130_g1_i1	Cuticle protein	7,107
c67686_g1_i1	γ-Hemocyanin	6,456	c65211_g11_i1	NADH 4	5,174	c64955_g2_i2	Arthrodial protein	6,799
c108471_g1_i1	NADH dehydrogenase	6,104	c50361_g2_i1	Unidentified protein	4,948	c898_g1_i1	NADH 1	6,743
c50494_g1_i1	Neuron protein	6,098	c65211_g1_i1	NADH 6	4,709	c62396_g1_i1	Oocyte finger protein	6,080
