# Supplementary material for: Candidate genes that have facilitated freshwater adaptation by palaemonid prawns in the genus Macrobrachium: identification and expression validation in a model species (M. koombooloomba)
Source: PeerJ. 2017 Feb 8;5:e2977. doi: 10.7717/peerj.2977 (PMC5301973; doi:10.7717/peerj.2977)
Supplement: Figure S1 — X-axis represents the number of blast matches while Y-axis shows the name of different species. [file peerj-05-2977-s003.pdf]

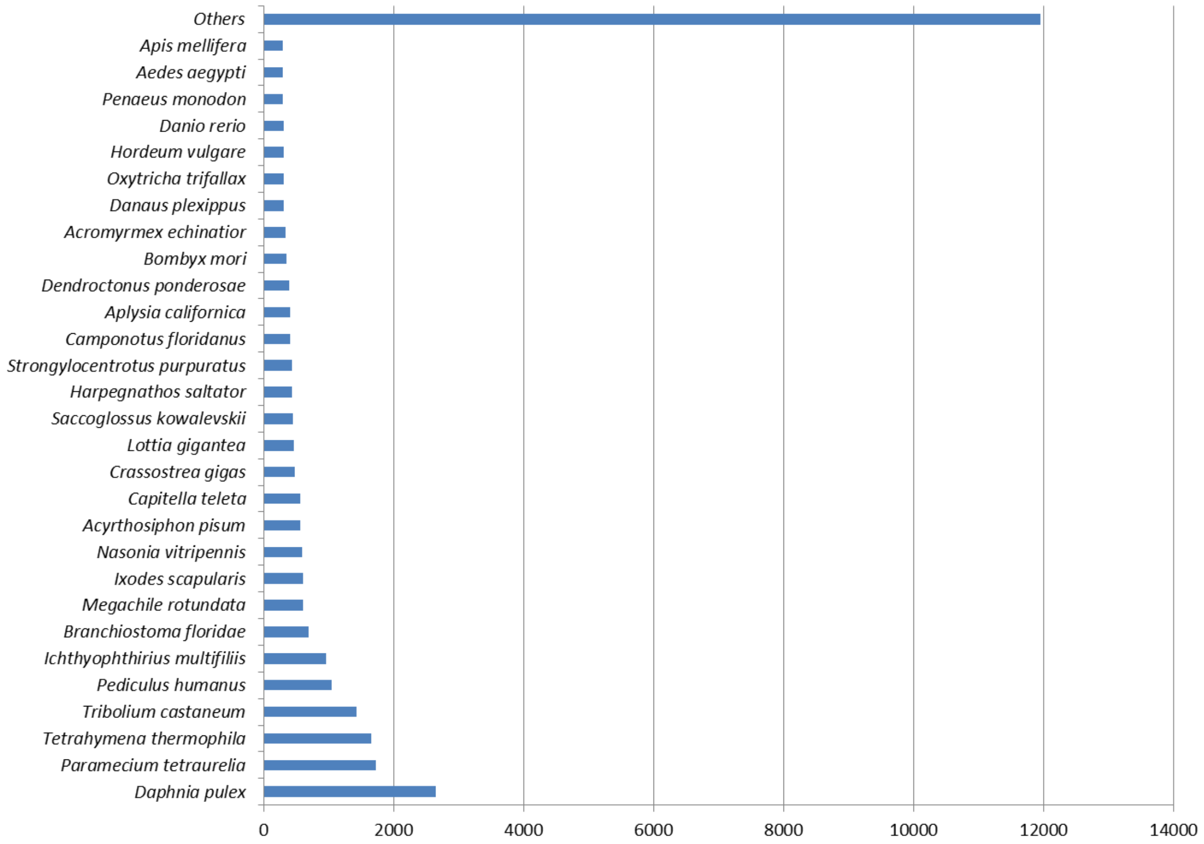

Figure S1: Top hit species distribution chart. X-axis represents the number of blast matches while Y-axis shows the name of different species.
