# Supplementary figures and images for: Candidate genes that have facilitated freshwater adaptation by palaemonid prawns in the genus Macrobrachium: identification and expression validation in a model species (M. koombooloomba)
Source: PeerJ. 2017 Feb 8;5:e2977. doi: 10.7717/peerj.2977 (PMC5301973; doi:10.7717/peerj.2977)

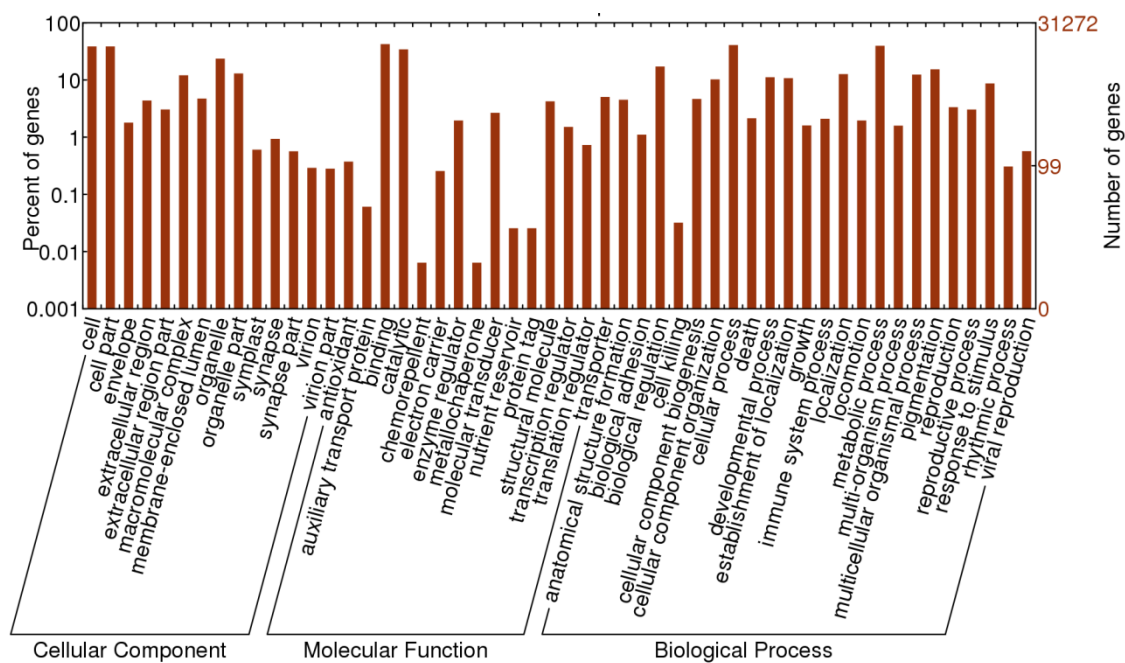

**Figure S2:** WEGO plot showing number and percentage of genes involved with different functions.

Supplement: Figure S2 [file peerj-05-2977-s004.pdf]
